# Antiviral Activity of Bay 41-4109 on Hepatitis B Virus in Humanized Alb-uPA/SCID Mice

**DOI:** 10.1371/journal.pone.0025096

**Published:** 2011-12-05

**Authors:** Nicolas Brezillon, Marie-Noëlle Brunelle, Hélène Massinet, Eric Giang, Céline Lamant, Lucie DaSilva, Sophie Berissi, Jacques Belghiti, Laurent Hannoun, Gherard Puerstinger, Eva Wimmer, Johan Neyts, Olivier Hantz, Patrick Soussan, Serban Morosan, Dina Kremsdorf

**Affiliations:** 1 INSERM, National Institute of Health and Medical Research, Unit 845, Paris, France; 2 Université Paris Descartes, Faculté de Médecine René Descartes, CHU Necker, Paris, France; 3 Institut Pasteur, Département de Virologie, Paris, France; 4 Department of Hepatobilary and Digestive Surgery, Hôpital Beaujon, Clichy, France; 5 Department of Hepatobilary and Digestive Surgery, Hôpital Pitié-Salpêtrière, Paris, France; 6 Department of Pharmaceutical Chemistry, Institute of Pharmacy, University of Innsbruck, Innsbruck, Austria; 7 Rega Institute for Medical Research, University of Leuven, Leuven, Belgium; 8 INSERM, National Institute of Health and Medical Research, Unit 871, Lyon, France; 9 Université Pierre et Marie Curie, Faculté de médecine Pitié-Salpêtrière, Centre d'Expérimentation Fonctionnelle, Paris, France; 10 Facultatea de Medicina Veterinara, Iasi, Romania; Institut Pasteur, France

## Abstract

Current treatments for HBV chronic carriers using interferon alpha or nucleoside analogues are not effective in all patients and may induce the emergence of HBV resistant strains. Bay 41-4109, a member of the heteroaryldihydropyrimidine family, inhibits HBV replication by destabilizing capsid assembly. The aim of this study was to determine the antiviral effect of Bay 41-4109 in a mouse model with humanized liver and the spread of active HBV. Antiviral assays of Bay 41-4109 on HepG2.2.15 cells constitutively expressing HBV, displayed an IC_50_ of about 202 nM with no cell toxicity. Alb-uPA/SCID mice were transplanted with human hepatocytes and infected with HBV. Ten days post-infection, the mice were treated with Bay 41-4109 for five days. During the 30 days of follow-up, the HBV load was evaluated by quantitative PCR. At the end of treatment, decreased HBV viremia of about 1 log(10) copies/ml was observed. By contrast, increased HBV viremia of about 0.5 log(10) copies/ml was measured in the control group. Five days after the end of treatment, a rebound of HBV viremia occurred in the treated group. Furthermore, 15 days after treatment discontinuation, a similar expression of the viral capsid was evidenced in liver biopsies. Our findings demonstrate that Bay 41-4109 displayed antiviral properties against HBV in humanized Alb-uPA/SCID mice and confirm the usefulness of Alb-uPA/SCID mice for the evaluation of pharmaceutical compounds. The administration of Bay 41-4109 may constitute a new strategy for the treatment of patients in escape from standard antiviral therapy.

## Introduction

More than 350 million people worldwide are chronically infected by hepatitis B virus (HBV), resulting in 500,000 to 1.2 million deaths/year from chronic hepatitis, cirrhosis or hepatocellular carcinoma (HCC) [1]. The therapies available for chronic hepatitis B infection are effective in reducing viremia and improving clinical outcomes, but no single therapy is optimal; each agent has its own benefits and drawbacks [2]. Long-term interferon alpha treatment is only effective in a third of patients and causes significant adverse effects such as fatigue, fever, muscle aches, bone marrow suppression, psychosis and autoimmune conditions [3]. Treatment with nucleos(t)ide analogues can enable a durable HBV DNA suppression of replication and an improvement in both hepatic fibrosis and hepatic decompensation [4]. However, the long-term use of such analogues may induce the emergence of drug-resistant HBV strains harboring mutations within the reverse transcription domain of the polymerase [5]. Alternative drug therapies, and investigation of their efficacy, are thus warranted. This requires the development of new agents that can block the viral life cycle at stages other than those associated with the viral polymerase, and target both wild-type and drug-resistant strains. During the past ten years, new drugs have been shown to disrupt HBV assembly by altering capsid formation. The chemical class of phenylpropenamide compounds can selectively inhibit HBV replication by acting at the level of pregenomic RNA packaging [6]. Alkylated imino sugars or Bis-ANS have been found to reduce the production of HBV by disrupting the maturation of HBV nucleocapsids [7], [8]. In the family of heteroaryldihydropyrimidines, Bay 41-4109 (methyl-4-(2-chloro-4-fluorophenyl)-2-(3,5-difluoro-2-pyridinyl)-6-methyl-1,4-dihdro-pyrimidine-5-corboxylate) has been identified as an effective inhibitor of HBV replication in cell cultures and in an HBV transgenic mouse model [9], [10]. It has been demonstrated, in vitro, that Bay 41-4109 was equally effective at inhibiting HBV DNA release and the cytoplasmic HBcAg level [11]–[15]. Bay 41-4109 acts in a capsid protein-specific manner throws the destabilization of the viral capsid nucleation by the formation of non-capsid polymers instead of nucleocapsid, preventing the formation of viral core particles [11]–[15]. In HBV transgenic mice, Bay 41-4109 caused a dose-dependent reduction of viral replication in liver and blood plasma and reduced core protein expression in the liver at the end of the treatment [9]. Preclinical studies for testing the pharmacokinetic and toxicity of Bay 41-4109 was performed on different animals and concluded to the suitability of the compound at concentrations from 3.3 to 50 mg/kg [11], [16].

Hepatocytes are some of the rare cells which have never successfully been cultivated for long periods in a differentiated form; so despite its undeniable value to *in vivo* study of the effects of viral protein expression in the liver, the transgenic mouse model is not fully satisfactory. Indeed, differences do exist (in terms of metabolic activity) between human and mouse hepatocytes. The lack of a small animal model susceptible to HBV infection has hampered the development of simple methods to evaluate new therapeutic compounds. In this context, we and others have developed a model of mice that are susceptible to HBV infection; the immunodeficient urokinase-type plasminogen activator (uPA/SCID) transgenic mouse, described as being a potent host for liver repopulation by human hepatocytes and HBV infection [17]–[19], [20], [21]. Human hepatocytes engrafted in the liver of uPA/SCID mice continue to express many of the human enzymes implicated in detoxification metabolism, so that the antiviral capacity of therapeutic molecules directed against hepatitis viruses can be assessed [18], [20]. Furthermore, by comparison with the HBV transgenic mouse model, the humanized uPA/SCID mouse model enables study of the impact of antiviral molecules during a complete cycle of HBV replication. The aim of the present study was therefore to assess the antiviral potential of Bay 41-4109 in humanized liver in a context of active viral spread.

## Materials and Methods

### Ethics Statement: Animals

The animals were kept under pathogen–free conditions and treated in accordance with European Union regulations on animal care (Directive 86/609/EEC). All procedures were approved by the local animal care (agreement A75-15-7-10) and use committee (“Comité de pilotage” of the faculty Necker).

### Transplantation of human hepatocytes and HBV infection

The generation of Alb-uPA/SCID mice has been described elsewhere [22]. Primary human hepatocytes were isolated from surgically collected liver-biopsy from patients undergoing therapeutic partial hepatectomy for liver metastasis (Department of Hepatobilary and Digestive Surgery from Beaujon and Pitié-Salpêtrière hospitals), in accordance with French ethical regulations (article L-1245-2 of the Huriet laws). The isolation of hepatocytes was performed by collagenase perfusion, as previously described [22]. Primary human hepatocytes were also obtained cryopreserved (Biopredic International, France). Preparations with at least 80% cell viability were transplanted (8×10^5^) into the spleen of 16–20-day-old uPA^+/+^/SCID mice [22]. The levels of human albumin in sera were quantified by ELISA, according to the manufacturer's instructions (Bethyl Laboratories).

HBV was concentrated from supernatants of the HepG2.2.15 line stably expressing HBV [23], [24]. The viral preparation was suspended in Williams's medium at a concentration of 1×10^10^ genome equivalents/ml. Four to five weeks after transplantation, animals with a level of human albumin secretion of at least 20 µg/ml were infected with 1×10^9^ HBV genome equivalents by intraperitoneal injection.

### Bay 41-4109 antiviral assays

Bay 41-4109 was synthesized as described elsewhere [25] and dissolved in a solution of 0.5% tylose (Fluka, Biochemika) and 1% ethanol. For the *in vitro* antiviral assay, HepG2.2.15 cells were seeded onto 6-well plates and treated with different concentrations of Bay 41-4109 (25 to 400 nM). The media were changed every two days and HBV DNA was analyzed in the supernatant five days post-plating (two days of viral secretion in the culture medium). The experiments were performed in duplicate. HBV-infected mice were treated 10 days post-infection. For the *in vivo* test, in accordance with previous data, the animals were fed with Bay 41-4109 (25 mg/kg body weight) twice a day for five days [9]. At appropriate time points, sera were harvested by retro-orbital bleeding. At the end of the experiment, the mice were sacrificed by cervical dislocation and their livers were recovered.

### HBV DNA quantification

HBV DNA was extracted from 400 µl of supernatant HepG2.2.15 or 20 µl of mouse sera as indicated by the manufacturer (Qiablood extraction kit, Qiagen, Courtaboeuf, France). HBV DNA quantification was performed by real-time PCR using HBV-specific primers and the LightCycler system (Roche-Applied-Science, France), as previously described [26]. For *in vitro* experiments, the IC50 was determined using GraphPad Prism 5 software. Statistical analyses were performed using the nonparametric Mann-Whitney test and GraphPad Prism 5 software. Data are represented as means ± standard deviation, and a p value<0.05 was considered to be significant.

### Immunohistochemistry

Liver biopsies were fixed in 10% formalin solution and embedded in paraffin. The sections were treated serially with either rabbit anti-α1 anti-trypsin (1∶2000, Dako, France) or rabbit anti-HBc (1∶500, Dako, France) antibodies. A secondary antibody conjugated with horseradish peroxidase (EN-VISION-kit; Dako) was then added. Sections were stained with diaminobenzidine and counterstained with hematoxylin (Dako, France).

## Results

### Bay 41-4109 inhibits the replication of HBV in the HepG2.2.15 cell line

**Figure 1:**
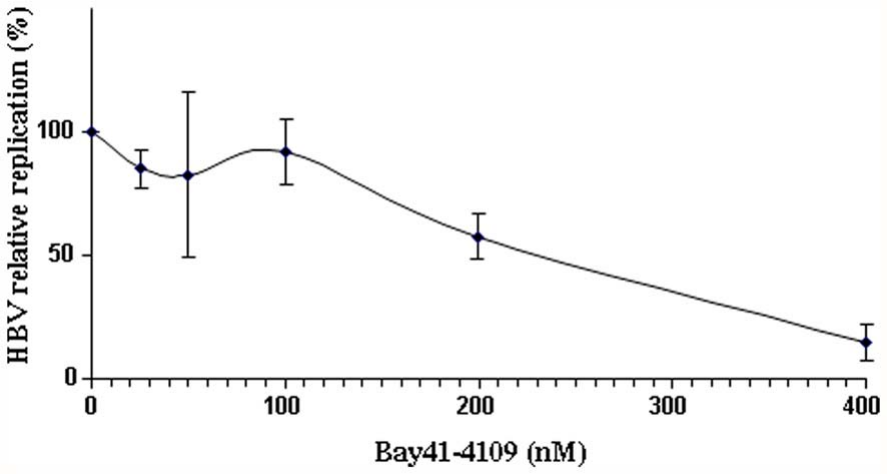
Antiviral activity of Bay 41-4109 on HBV replication in HepG2.2.15 cells. Cells were treated with Bay 41-4109 (25, 50, 100, 200 and 400 nM) as described in the Methods section. HBV DNA in HepG2.2.15 supernatant was quantified by real-time PCR. The data represent the results of three independent experiments, performed in duplicate.

It had previously been reported that in HepG2.2.15 cells stably producing HBV, only (-)R-enantiomers of Bay 41-4109 were active against HBV [25]. Knowing that our batch of Bay 41-4109 was a 1/1 racemic mixture, we decided to assay the inhibitory concentration of the batch that was required to decrease HBV replication by 50% (IC_50_). To achieve this, the HepG2.2.15 cell line was treated for five days with different concentrations of Bay 41-4109. The ability of Bay 41-4109 to inhibit HBV replication was then analyzed using real-time PCR quantification of extracellular HBV DNA (Fig. 1). The IC_50_ of our Bay 41-4109 preparation was about 202 nM. In addition, no cell toxicity was evidenced alongside its efficacy against HBV replication. Indeed, at the end of the five days of treatment not cell death was observed. Furthermore, microscopic analysis of the cells did not evidenced markers of cell stress. This batch of Bay 41-4109 was therefore used for our *in vivo* studies.

### Bay 41-4109 diminishes HBV viremia in humanized Alb-uPA/SCID mice

The *in vivo* antiviral effects of BAY 41-4109 on HBV expression had previously been investigated in a transgenic mouse model that mimics chronic HBV infection [9]. Because at the beginning of infection our model enabled the observation of active viral replication, we decided to evaluate the impact of a short period of treatment. Primary human hepatocytes were transplanted into uPA homozygous mice, and four to five weeks later mice with circulating levels of human albumin of at least 20 µg/ml were infected with HBV.

**Figure 2:**
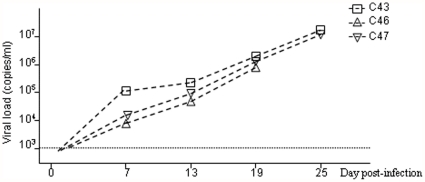
Kinetics of HBV replication in humanized Alb-uPA/SCID mice. The HBV viral load during the course of the experiment was quantified by real-time PCR in sera from three mice.

**Figure 3:**
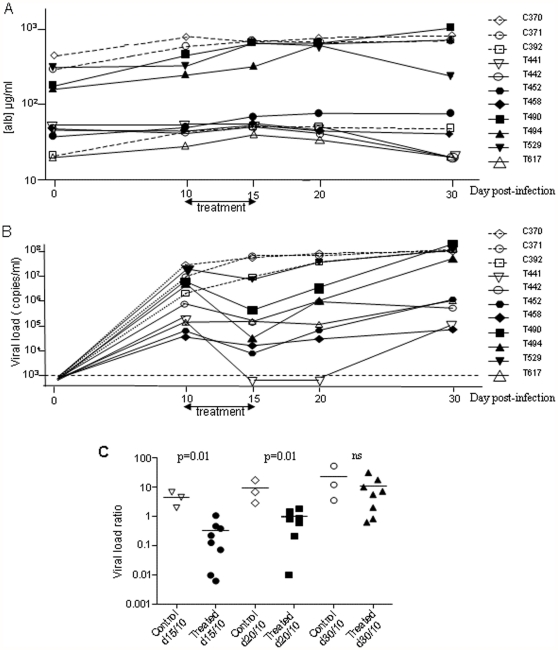
Antiviral activity of Bay 41-4109 on HBV replication in humanized Alb-uPA/SCID mice. (A) Human albumin concentrations in sera from treated (full lines) and untreated (dotted lines) animals. (B) The HBV viral load during the course of the experiment was quantified by real-time PCR in sera from treated (full lines) and untreated (dotted lines) animals. (C) Histograms represent the mean HBV load ratio at specific time points in each treated (white) and untreated (black) animal. Data are represented on semilogarithmic graphs.

In order to perform the treatment during the active phase of HBV replication in our model, we first followed viral replication for 19 or 25 days post-infection. In the three animals analysed, a sustained increase in the HBV viral load was observed at all the time points tested (Fig. 2). A total of eleven chiremic mice with circulating levels of human albumin of at least 20 µg/ml (mean 121 µg/ml, range: 20–450 µg/ml) were infected with HBV. Eight of these eleven animals were treated with Bay 41-4109 for five days, between day 10 and day 15 post-infection (Fig. 3). During the 30-day follow-up period, only minor variations in human albumin levels were observed (Fig. 3A). This seemed to indicate an absence of toxicity of Bay 41-4109 on human hepatocytes, at least at the concentrations used and during the period of application in this experiment.

HBV viral loads were determined by real-time PCR in the sera of infected mice at 10, 15, 20 and 30 days post-infection (Fig. 3B and C, Table 1). On the day of treatment (D10) the HBV viral load ranged from 4.57 to 7.48 log(10) copies/ml and no significant differences were observed between the untreated and treated groups (Fig. 3B, Table 1). At the end of the five days of treatment (D15), all but one of the treated mice displayed a decrease in HBV viral replication (mean decrease: about 1 log(10) copies/ml); at the same time, an expansion of HBV replication (mean increase: about 0.5 log(10) copies/ml) was evidenced in untreated mice. Furthermore, at the end of treatment (D15) and five days later (D20), the HBV viral load differed significantly between the treated and untreated groups. It should be noted that the reduction in HBV viral load was not correlated to the initial viremia. In one treated animal (T441), the HBV viral loads were below the level of detection at both time points.

**Table 1:** HBV viral load in control and treated infected uPA chemeric mice.

	Day post-infection			
mice	10(Log copies/ml)	15(Log copies/ml)	20(Log copies/ml)	30(Log copies/ml)
control				
C370	7.48	7.78	7.90	8.00
C371	6.99	7.85	7.85	8.08
C391	6.32	6.98	7.60	8.05
Mean±SD	6.9±0.6	7.53±0.48	7.78±0.16	8.04±0.04
Treated				
T441	4.98	<2.00^1^	<2.00^1^	4.64
T442	5.82	5.16	5.99	5.73
T452	4.81	3.91	4.84	6.07
T458	4.57	4.23	4.49	4.87
T490	6.80	5.65	6.57	8.30
T494	6.71	4.52	6.03	7.70
T529	7.30	6.88	7.60	8.00
T617	5.17	5.20	5.08	6.03
Mean±SD	5.86±1.04	4.62±1.43	5.36±1.68	6.47±1.41
p^2^	ns	0.01	0.02	ns

1 an arbitrary threshold of detection of 100 copies/ml was used for calculation.

2 nonparamertic Mann-Whitney test.

To further validate our data we decided to evaluate the impact of Bay 41-4109 treatment independently of the initial level of HBV replication. For this purpose, the ratio between the pre-treatment HBV load and that seen at specific time points after treatment was compared in the two groups (Fig. 3C). Under these conditions, the specificity of the HBV viral load decrease in the treated group at D15 and D20 was confirmed.

**Figure 4:**
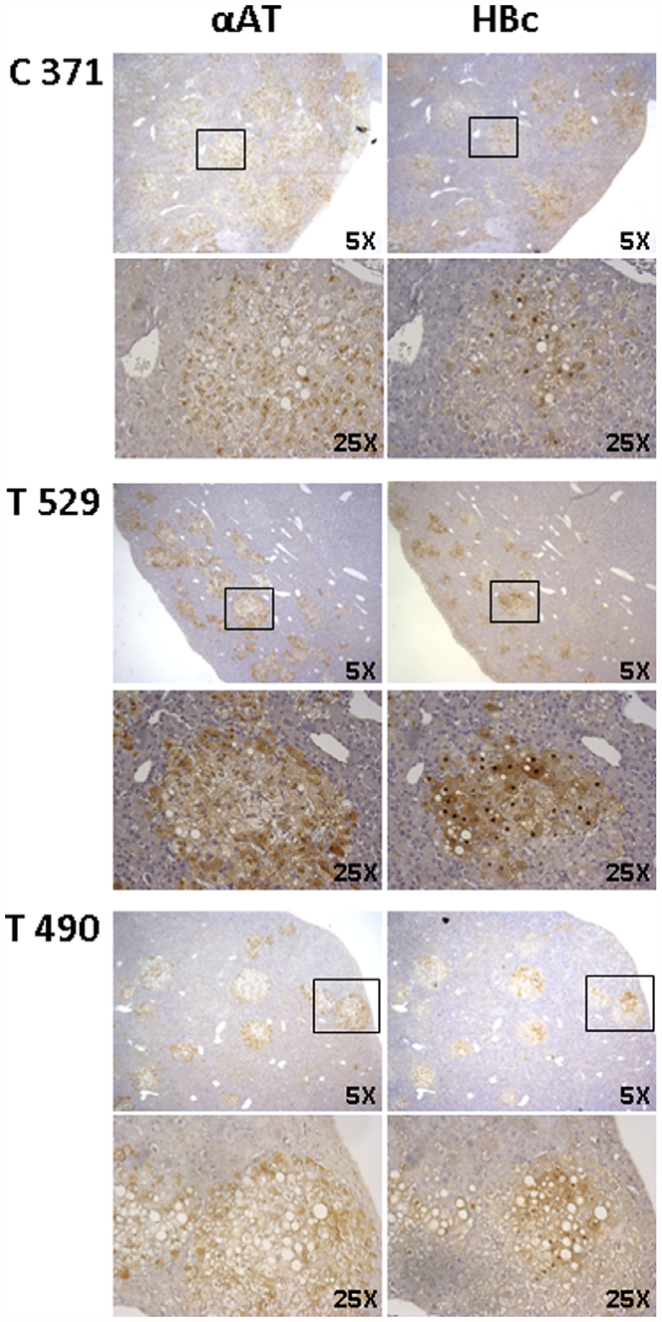
Immunohistological analysis of human hepatocytes in mouse livers. Serial sections of chimeric mouse livers from treated (T529 and T490) and untreated (C371) mice were analyzed by labeling with anti-α1 anti-trypsin (αAT) or with anti-HBc (HBc) antibodies.

Finally, 10 days later (D30), a viral rebound was measured in all mice in the treated group, to the extent that there were no longer any significant differences between the HBV viral loads of the two groups. In accordance with these data, immunohistochemistry assays in the humanized nodules 30 days after infection demonstrated a similar level of capsid expression, independently of treatment (Fig. 4). In addition, for treated mice, examination of liver section did not showed any evidences of liver lesions.

## Discussion

Previous *in vitro* reports have demonstrated that the antiviral mechanism of Bay 41-4109 against HBV was the result of an inhibition of capsid assembly. However, *in vivo*, the antiviral efficacy of Bay41-4109 was only described in an HBV transgenic mouse model. In this mouse model, however, HBV replication is not complete (absence of cccDNA formation and an infection step) and the metabolic context of a mouse liver is less relevant. Indeed, the detoxification pathway implicated in the metabolism of drugs is highly specific to each species, so that a humanized liver becomes even more appropriate. The generation of uPA-SCID mice with a humanized liver constitutes a major advance in the study of human hepatotropic viruses. Transplanted human hepatocytes can thus reside in their natural environment and maintain normal functions. The usefulness of the humanized mice model has been demonstrated when evaluating the efficacy of antiviral compounds [27]. The goal of the present study was to determine whether the Bay41-4109 molecule retained its antiviral properties in a human context during a complete active phase of HBV replication.

Our study therefore used mice with a low level of human albumin secretion, as we had previously observed that the level of human hepatocytes in mouse liver was not strictly correlated to the level of HBV viral replication. This lack of a strict correlation between HBV infection efficacy and human albumin levels has already been reported elsewhere [28].

In the humanized context of our mouse model, we showed that five days of treatment with Bay 41-4109 were sufficient to reduce HBV replication, with a mean decrease greater than 1 log (10). These results support and extend previous findings obtained in HBV transgenic mice after 28 days of treatment [9]. Furthermore, our results demonstrate that Bay 41-4109 conserved its antiviral efficacy against HBV replication, even in a context of virus spread, and we demonstrated that five days of treatment with Bay 41-4109 was sufficient to reduce HBV replication without causing the loss of a human hepatocyte graft. This was in agreement with previous data showing that in rat rats treated with different doses of Bay 41-4109 changes to hepatotoxicity only occurred at high-doses (≥100 mg/kg/d) [16].

Despite the clear impact of this treatment, a short course was insufficient to abrogate viral replication permanently. Indeed, five days after treatment discontinuation, we observed a rebound of HBV replication, which was confirmed at the end of the experimental period. Nevertheless, a control of viral spread was evidenced, together with an absence of progression of the viral load. The administration of Bay 41-4109 for a longer period of time in this mouse model will permit an assessment of the long-term toxicity of the compound and its ability to permanently abolish viral replication.

In conclusion, our findings demonstrate that the Alb-uPA/scid model provides a valuable bridge between the preclinical screening of pharmaceutical compounds and clinical assessment of their efficacy and toxicity in human. It should be notified that a phase I pre-clinical assay is under evaluation, but until now, no clear data is yet available. Nevertheless, our observations emphasize the usefulness of Bay 41-4109 as a valuable supplement to current therapies for HBV infection, and it could be tested as a curative tool during the spread of HBV resistant strains that occurs during treatment with nucleoside analogues.
